# Challenges in diagnosing and monitoring radiographic and non-radiographic axial spondyloarthritis in daily clinical practice

**DOI:** 10.1007/s10067-026-08157-0

**Published:** 2026-05-14

**Authors:** Anneke Spoorenberg, Yvonne Maria van der Kraan, Maria de Hair, Suzanne Arends

**Affiliations:** 1 Department of Rheumatology and Clinical Immunology, University Medical Center Groningen, Groningen, The Netherlands; 2 Novartis Pharma BV, Amsterdam, The Netherlands

**Keywords:** Axial spondyloarthritis, Clinical decision-making, Clinical guidelines, Disease activity assessment, Survey

## Abstract

**Introduction/objectives:**

Axial spondyloarthritis (axSpA) is a heterogeneous disease with variable clinical features changing over the disease course, making diagnosis and monitoring challenging. Large progress has been made in classification development, imaging, disease-activity assessment. However, there is limited insight in how these developments are addressed in daily clinical practice. Therefore, our study aim was to gain better insight into diagnosis, monitoring and treatment and to explore if non-radiographic (nr-)axSpA is addressed differently from radiographic (r-)axSpA.

**Methods:**

A multiple-choice survey on diagnosis, monitoring, and treatment of axSpA was conducted in semi-structured, face-to-face interviews with 51 Dutch rheumatologists, representing a range of geographical locations, hospital types, and axSpA expertise.

**Results:**

Of all participating rheumatologists, 78% worked in secondary referral centers, reflecting Dutch rheumatology practice. 52% felt insufficiently skilled to independently assess MRI sacroiliitis. Diagnostic uncertainty was higher for nr-axSpA (23%) than r-axSpA (10%). ASAS classification criteria were used to diagnose nr- and r-axSpA by 54% and 36% of rheumatologists. For monitoring disease activity, ASDAS was always or never used by 28% and 26% of rheumatologists. In treatment decisions concerning biological DMARDs, the level of pain was considered equally important as disease activity scores (ASDAS, BASDAI). Most rheumatologists (60%) did not use ASDAS or BASDAI cut-off scores or change to evaluate biological DMARDs’ effectiveness.

**Conclusions:**

This study reveals that Dutch rheumatologists experienced difficulties in diagnosing axSpA in one out of four patients. ASAS classification criteria were often used for support, particularly in diagnosing nr-axSpA. The use of disease-activity assessments during monitoring was limited.

**Table:** 

**Key Points** •*Diagnostic uncertainty remains common in daily practice, particularly for non-radiographic axial spondyloarthritis.* •*Although ASAS classification criteria were developed for research purposes, they are also used to support diagnosis.* •*ASDAS is used inconsistently to monitor disease activity and many rheumatologists seem to base treatment decisions on reported pain level.* •*There seems to be a gap between axial spondyloarthritis international management recommendations and routine rheumatology care.*

**Supplementary Information:**

The online version contains supplementary material available at 10.1007/s10067-026-08157-0.

## Introduction

Axial spondyloarthritis (axSpA) is a heterogeneous auto-inflammatory rheumatic disorder of pre-dominantly the sacroiliac (SI) joints and the spine [1]. The key symptom is chronic (low) back pain, typically beginning before the age of 45 [1]. Additional features of axSpA are an inflammatory character of the back pain with a good response to NSAIDs, evidence of sacroiliitis on conventional radiographs or SI magnetic resonance imaging (MRI), the presence of the human leukocyte antigen (HLA)-B27, peripheral manifestations (such as arthritis, dactylitis, and enthesitis), extra-musculoskeletal manifestations (EMM), including uveitis, psoriasis, and inflammatory bowel disease, a family history of SpA or EMM. Also, C-reactive protein (CRP) may be elevated in case of active disease. However, axSpA features may be limited and vary over time and since none of the features are specific for axSpA, diagnosis is made based on clinical picture [1].

Before MRI became available to demonstrate active sacroiliitis, ankylosing spondylitis, nowadays radiographic axSpA (r-axSpA), was the only recognized subtype of axSpA, with radiographic sacroiliitis on pelvic X-rays considered mandatory for diagnosis [2]. However, in the past two decades, MRI has emerged as a more sensitive tool to detect early inflammatory lesions in patients with chronic back pain and other axSpA features but without radiographic sacroiliitis [3]. As a result, it was proposed that these patients have the same underlying disease as those with r-axSpA, leading to the definition of non-radiographic axSpA (nr-axSpA) [3]. Despite advances in imaging, the diagnosis of axSpA remains challenging due to the large heterogeneity in clinical presentation and evolving nature of the disease. Diagnosis is particularly difficult in the early stages when features may not have fully developed [4]. Primarily for research purposes, the Assessments for SpondyloArthritis International Society (ASAS) developed classification criteria, established in 2009, which include both r-axSpA and nr-axSpA [5, 6]. The criteria are based on chronic back pain lasting at least 3 months, beginning before the age of 45. Patients meet the criteria via either the imaging arm, which requires evidence of sacroiliitis (on conventional radiographs of the pelvis or MRI of the SI joints) along with at least one additional SpA feature, or the clinical arm, which requires the presence of HLA-B27 and at least two other SpA features. However, a positive classification does not necessarily confirm an axSpA diagnosis, as clinical judgment based on pattern recognition remains essential [7].

Besides diagnosing, also monitoring axSpA is challenging, as physical examination of the axial skeleton is often normal, especially in early disease [8]. Furthermore, disease activity assessments rely largely on patient-reported outcomes (PROs) [9, 10]. The Bath Ankylosing Spondylitis Disease Activity Index (BASDAI) was first preferred to monitor disease activity until the development of the Axial Spondyloarthritis Disease Activity Score (ASDAS) in 2009 [11]. ASDAS is currently recommended in both research and daily clinical practice. ASDAS is an algorithm incorporating scores from three of the six BASDAI questions (axial pain, peripheral pain/inflammation, duration of morning stiffness), patient global disease activity, and CRP level [10, 12]. Although ASDAS has better psychometric properties than BASDAI, CRP is elevated in only up to 40% of axSpA patients with inflammatory disease, limiting the ability to objectively assess inflammation in all patients [13]. Monitoring the disease includes a combination of clinical findings, CRP, imaging, and PROs, including the disease activity assessments ASDAS and BASDAI [8, 14]. Repeating conventional radiography of the SI joints and the spine is not recommended within the first 2 years due to overall slow progression of radiographic damage such as the development of syndesmophytes [8, 14]. Also, MRI of the SI joints and spine is still not recommended for routine monitoring due to its high cost and uncertainty about utility, although randomised controlled clinical trials (RCTs) with biological- and targeted synthetic disease-modifying anti-rheumatic drugs (b/tsDMARDs) have shown a significant decrease in inflammatory lesions on MRI [8, 14–16].

Management recommendations for axSpA have been updated in 2016 and 2022 [14, 17]. The overarching principles remained unchanged [14, 17]. Patients should be educated about the disease and associated lifestyle aspects, for example, healthy diet and weight management, encouraged to exercise, and advised to quit smoking [14]. NSAIDs are still the first-line pharmacological treatment option for pain and stiffness in axSpA [14]. If NSAIDs fail to control disease activity, more effective pharmacological treatment options have become available in the past two decades. In chronological order the following types of b/tsDMARDs have become available: TNF inhibitors (TNFi), IL-17 inhibitors (IL-17i), and JAK inhibitors [14]. As a first prescription, TNF inhibitors (TNFi) or IL-17 inhibitors (IL-17i) have a slight preference due to longer experience. So far, all b/tsDMARDs show similar efficacy in RCTs although there are only a few head-to-head comparisons [14]. After b/tsDMARD failure, switching to another bDMARD or tsDMARD should be considered [14]. The 2022 update introduced two additional recommendations to the 2016 recommendations. Firstly, consider total hip arthroplasty for patients with refractory pain or disability and structural damage, irrespective of age, and spinal corrective osteotomy may be an option for severe disabling deformity in specialized centers [14]. Secondly, it is recommended that, if there is a ‘substantial change in the disease course,’ non-inflammatory causes like spinal fractures be considered and appropriate evaluation, including imaging, be performed [14]. There is still limited information on how these developments in classification, diagnosis, monitoring, and treatment of axSpA over the past two decades are addressed by rheumatologists in daily clinical practice. Therefore, the aim of this exploratory study was to gain insight into the axSpA patient’s journey regarding diagnosis, monitoring and treatment in Dutch rheumatology practices, and to explore if nr-axSpA patients are addressed differently from r-axSpA patients.

## Materials and methods

### Participants

Rheumatologists in the Netherlands were invited to participate in a survey concerning diagnosis, monitoring and treatment of patients with axSpA in their daily clinical practice. The survey targeted a diverse group of rheumatologists from different geographical regions, hospital types (secondary-referral centers and university hospitals), and included both axSpA experts and non-experts.

### The survey

The study and survey were designed by the whole research team, led by AS and SA. It consisted of 37 multiple-choice questions, beginning with a brief introduction outlining the survey’s aim and structure (Supplementary file 1). Data collection was performed by representatives of the medical department of Novartis Netherlands (MH, MS, RB) through face-to-face, semi-structured interviews. During these interviews, the interviewer read each question verbatim together with the predefined multiple-choice response options, and responses were recorded on paper. Interviewers did not systematically probe or ask follow-up questions beyond clarification when requested by the respondent. Subsequently, these responses were entered in ‘I-Journey’, a survey platform by ‘14eight’. Rheumatologists were financially compensated for the interviewing time according to national compliance guidelines of the Dutch government. Data analysis was conducted independently by AS, YK, and SA. Interpretation of the results and manuscript drafting were primarily performed by AS and YK, with input and critical revision from all authors. This study had no commercial objectives. The study was conducted in accordance with the Declaration of Helsinki. Prior to the interview, participants gave their approval for the anonymous use of the data and its potential publication.

#### General information

The first part of the questionnaire collected general information, including years of experience as a rheumatologist, type of clinic (general hospital, private care center, or university hospital), and an estimate of the number of axSpA patients treated in their practice.

#### Referral and diagnosis

The second part of the survey included multiple-choice questions about referral and diagnosis. For referral, participants were asked about the percentage of patients with possible axSpA referred by general practitioners, ophthalmologists, gastroenterologists, dermatologists, or other health professionals (totalling 100%), as well as the symptom duration before referral (< 3 months, 3–6 months, 7–12 months, 1–2 years, or > 2 years). Questions about diagnosis were addressed separately for nr-axSpA and r-axSpA. Participants rated the importance of various patient characteristics and axSpA-related features for diagnosis on a 5-point numerical rating scale (very unimportant, unimportant, neutral, important, very important), including male sex, onset of back pain at ≤ 45 years of age, inflammatory back pain, back pain improving at least 50% with full-dose NSAIDs, presence of a peripheral manifestation (arthritis, dactylitis, enthesitis), and presence of an EMM (uveitis, inflammatory bowel disease, psoriasis). Next, participants indicated the percentage of patients for whom they used conventional pelvic radiographs, MRI of the SI joints, HLA-B27 testing, and CRP/ESR for diagnostic purposes. They were also asked whether a musculoskeletal (MSK) radiologist was available to review imaging, whether they encountered uncertainty about the accuracy of radiology reports, and whether they felt sufficiently skilled to evaluate MRI images of the SI joints themselves. Finally, participants reported whether they used the modified New York (mNY) criteria (for r-axSpA only), ASAS classification criteria (for both nr- and r-axSpA), or no criteria at all to help them confirm the diagnosis, and the percentage of patients in whom they experienced uncertainty in confirming the final diagnosis.

#### Monitoring and treatment

The third part of the survey focused on monitoring and treatment. Participants were asked about several aspects, including the percentage of axSpA patients referred to a nurse practitioner for patient education (0%, 1–25%, 26–50%, 51–75%, 76–100%) and the topics covered during education sessions (general information about axSpA, prognosis, exercise therapy and sports, and lifestyle factors such as smoking, diet, work, and stress). They were also asked about the percentage of patients referred to a physiotherapist for exercise therapy (0%, 1–25%, 26–50%, 51–75%, 76–100%), the availability of multidisciplinary consultations with ophthalmologists, dermatologists, and gastroenterologists, referrals for multidisciplinary rehabilitation programs, and the percentage of patients referred to a nurse practitioner as a substitution of care (under rheumatologist supervision). Questions about disease activity monitoring addressed the types of assessments used (ASDAS, BASDAI, CRP/ESR) and their frequency of use (always, often, sometimes, never). Regarding pharmacological treatment, participants indicated the percentage of patients on a full dose of NSAIDs before and after starting a bDMARD. Additionally, they rated the importance of various disease activity measures and clinical features in the decision to initiate a bDMARD on a 5-point numerical rating scale (very unimportant to very important). Items assessed included lack of response after two NSAIDs for at least 4 weeks, ASDAS > 2.1, BASDAI > 4, experienced pain related to axSpA, elevated CRP (> 5 mg/L), presence of peripheral manifestations (arthritis, enthesitis, dactylitis), presence of EMM (uveitis, inflammatory bowel disease, psoriasis), signs of inflammation on MRI of the SI joints or spine (e.g., bone marrow edema), axSpA-related structural damage on radiographs, and male sex. Finally, participants were asked whether they used the 2016 ASAS-EULAR treatment recommendations for axSpA as guidance in clinical practice (yes, no, or not familiar).

### Statistical analysis

Statistics were performed using IBM SPSS 23.0 software for Windows (SPSS, Chicago, IL, USA). Descriptive statistics were applied for all relevant variables; results were expressed as percentages (%) for categorical data and mean ± standard deviation (SD) or median (interquartile range (IQR)) for normally distributed and non-normally distributed data, respectively. Comparisons of assessments and the perceived importance of patient- and disease characteristics contributing to the diagnosis or the decision to initiate a bDMARD between r-axSpA and nr-axSpA were conducted using the Wilcoxon signed-rank test. *p*-values ≤ 0.05 were considered statistically significant.

## Results

### General information

In total, 51 rheumatologists participated in the survey between October 2019 and November 2020, representing approximately 22% of all practicing rheumatologists in the Netherlands. The mean interview time was one hour. Due to the COVID pandemic, the final seven interviews were conducted via Microsoft Teams. The rheumatology clinics were evenly spread over the geographic regions of the Netherlands, mostly secondary-referral centers (78%). Eight (16%) rheumatologists had a special interest in axSpA. Most rheumatologists (80%) monitored more than 200 patients with axSpA in their outpatient clinics, with the majority diagnosed with r-axSpA. Furthermore, most rheumatologists (42%) reported diagnosing three to five axSpA patients every month, with an even distribution between nr- and r-axSpA. All respondent characteristics are presented in Table 1.

**Table 1:** Characteristics of rheumatologists (*n* = 51) and axSpA clinics

*Type of rheumatology clinic*		
Tertiary referral center		7 (14%)
Secondary referral center		40 (78%)
Independent treatment center		4 (8%)
*N *(%)* of patients with axSpA monitored in outpatient clinics*		
0–200		10 (20%)
201–400		17 (34%)
401–600		4 (8%)
> 600		9 (18%)
Unknown		4 (8%)
Percentage patients with nr-axSpA		
0–20%		11 (22%)
21–40%		21 (42%)
41–60%		13 (26%)
61–80%		2 (4%)
81–100%		0 (0%)
Unknown		3 (6%)
*N* (%) *of newly diagnosed patients with axSpA per month*		
0–2		16 (32%)
3–5		21 (42%)
6–10		9 (18%)
> 10		9 (18%)
Unknown		2 (4%)
Percentage nr-axSpA diagnosis		
0–20%		11 (22%)
21–40%		9 (18%)
41–60%		18 (36%)
61–80%		7 (14%)
81–100%		3 (6%)
Unknown		2 (4%)
*N* (%) *of patients with axSpA treated with bDMARD*		
	r-axSpA	nr-axSpA
0–10%	1 (2%)	7 (14%)
11–20%	3 (6%)	12 (24%)
21–30%	10 (20%)	13 (26%)
31–40%	12 (24%)	7 (14%)
41–50%	17 (34%)	6 (12%)
> 50%	4 (8%)	1 (2%)
Unknown	3 (6%)	4 (8%)
*Referring medical professional*		
General practitioner		70% [50–80%]
Ophthalmologist		5% [2–10%]
Gastroenterologist-hepatologist		10% [5–16%]
Dermatologist		5% [3–10%]
Other		5% [0–15%]
*Symptom duration before referral*		
< 3 months		0% [0–5%]
3–6 months		10% [1–20%]
7–12 months		15% [10–40%]
1–2 years		20% [12–40%]
> 2 years		30% [10–53%]
*Time (week) between referral and first outpatient visit*		
< 1		7 (14%)
1–2		8 (16%)
2–6		9 (18%)
> 6		26 (52%)

Values are presented in *n* (%) or median [IQR]. *axSpA* axial spondyloarthritis, *nr-axSpA* non-radiographic axial spondyloarthritis, *bDMARD* biological disease-modifying antirheumatic drug, *r-axSpA* radiographic axial spondyloarthritis

### Referral and diagnosis

#### Referral

Most patients (70%) were referred by the general practitioner. Approximately 50% had a symptom duration of more than 1 year, and 30% had more than 2 years. For most patients, the time between referral and the first outpatient clinic visit exceeded 6 weeks. Findings regarding referral are presented in Table 1.

#### Diagnosis

**Fig. 1:**
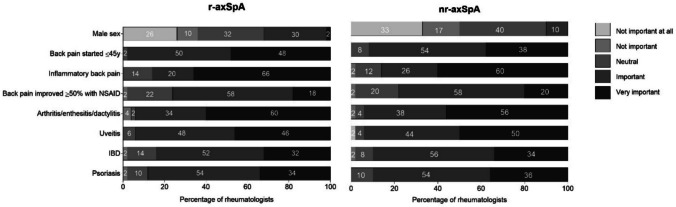
Level of importance of disease activity assessments and other disease features contributing to the r-axSpA/nr-axSpA diagnosis according to rheumatologists

Concerning the axSpA-related symptoms and features, back pain starting at ≤ 45 years of age, inflammatory back pain, back pain improving at least 50% on full dose of NSAIDs, the presence of peripheral manifestations (arthritis, dactylitis, enthesitis), and the presence of EMM were all considered important contributors to the axSpA diagnosis. Male sex was considered a less important contributor to the diagnosis. The level of importance of these features was overall the same for r-axSpA and nr-axSpA. However, male sex (*p* < 0.001) and back pain starting at ≤ 45 years of age (*p* = 0.011) added significantly more to the diagnosis of r-axSpA than nr-axSpA (*p *< 0.001 and *p* = 0.011, respectively). Figure 1 presents the findings on how various disease features contribute to the diagnosis of both r-axSpA and nr-axSpA.

In almost all patients in whom axSpA was considered, conventional X-pelvis were performed, and HLA-B27 status and CRP/ESR were assessed. MRI-SI was significantly more often performed to diagnose nr-axSpA compared to r-axSpA, in 93% and 20%, respectively. Most rheumatologists (80%) had access to an MSK radiologist. In approximately 15% of all MRI-SI reports made by radiologists, rheumatologists were uncertain about the accuracy, especially in cases where no MSK radiologist was available. More than half (52%) of the rheumatologists felt they were insufficiently equipped to determine signs of active sacroiliitis or damage on MRI. In 23% and 10% of patients with suspected nr-axSpA and r-axSpA, respectively, rheumatologists were uncertain about the clinical presentation, which made it difficult to establish a definitive axSpA diagnosis. In these cases, most rheumatologists opted to monitor symptom developments over time. For diagnosing r-axSpA, 28% of rheumatologists used the mNY criteria, 36% used the ASAS classification criteria and the remaining 36% did not rely on any criteria. To diagnose nr-axSpA, 54% of rheumatologists used the ASAS classification criteria and the remaining 46% did not use any criteria. All rheumatologists with special interest in axSpA did not use specific criteria for diagnosis. See Table 2 for all findings regarding the clinical investigations and criteria used by rheumatologists in diagnosing axSpA.

**Table 2:** Clinical investigations and criteria used by rheumatologists in diagnosing axSpA

*Additional clinical investigations*	r-axSpA	nr-axSpA
X-pelvis	100% [100–100%]	100% [100–100%]
HLA-B27	100% [50–100%]	100% [79–100%]*
CRP/ESR	100% [100–100%]	100% [100–100%]
MRI-SI	20% [4–51%]	93% [60–100%]*
*Criteria*		
mNY	14 (28%)	NA
ASAS	18 (36%)	27 (54%)
No criteria	18 (36%)	23 (46%)

*Statistically significant at *p* < 0.05

Values are presented in median [IQR] or *n* (%). *axSpA* axial spondyloarthritis, *r-axSpA* radiographic axial spondyloarthritis, *nr-axSpA* non-radiographic axial spondyloarthritis, *X-pelvis* radiograph of the pelvis, *HLA-B27* human leukocyte antigen B27, *CRP* C-reactive protein, *ESR* erythrocyte sedimentation rate, *MRI-SI* magnetic resonance imaging of the sacroiliac joints, *mNY* modified New York, *NA* not applicable, *ASAS* assessments for spondyloarthritis international Society

### Monitoring and treatment

#### Monitoring

After diagnosing axSpA, the majority (72%) of rheumatologists referred more than 75% of their axSpA patients to a nurse practitioner for patient education. General information about the disease was always discussed, and disease prognosis was addressed in 57% of patients. The role of exercise therapy and sports was discussed in 98%, and other lifestyle aspects such as smoking and diet in 88% of patients. Most rheumatologists (82%) referred at least 50% of their axSpA patients to a physiotherapist for exercise therapy. In most rheumatology practices (66%), there were no regular or scheduled multidisciplinary consultations with dermatologists, gastroenterologists or ophthalmologists to discuss overlapping treatment options in case of EMM. However, informal consultations with dermatologists were reported most often (32%), depending on the type of EMM. Most rheumatologists (76%) referred up to 25% of axSpA patients to a rehabilitation center for a multidisciplinary trajectory at some point during the disease course. During follow-up, 66% of rheumatologists referred their patients with axSpA to the clinical care of a nurse practitioner. Regarding the role of imaging, radiographs of the SI joints were performed during follow-up in 75% of patients with axSpA. Radiographs of the spine were conducted in 65% of patients. MRIs of the SI joints and spine were less frequently performed, in 20% and 5% of patients, respectively. Bone mineral density scans were obtained in 20%, and serum vitamin D levels were assessed in 28% of patients. All findings regarding management and clinical assessments during follow-up are presented in Table 3.

**Table 3:** Management and clinical assessments during follow-up of axSpA patients

*N *(%) *of patients with axSpA referred for patient education*	
0%	1 (2%)
1–25%	4 (8%)
26–50%	6 (12%)
51–75%	3 (6%)
76–100%	36 (72%)
*Patient education subjects*	
General information about axSpA	49 (100%)
Prognosis of axSpA	28 (57%)
Role of exercise therapy/sports	48 (98%)
Effect of other lifestyle aspects, e.g., smoking and diet	43 (88%)
*N (%) of patients with axSpA referred to physiotherapist for exercise therapy*	
0%	0 (0%)
1–25%	3 (6%)
26–50%	6 (12%)
51–75%	11 (22%)
76–100%	30 (60%)
*N (%) of rheumatologist with regular multidisciplinary consultancies with:*	
Dermatologist	16 (32%)
Gastroenterologist-hepatologist	3 (6%)
Ophthalmologist	0 (0%)
No multidisciplinary consultancies	33 (66%)
*N (%) of patients with axSpA referred for multidisciplinary rehabilitation*	
0%	6 (12%)
1–25%	38 (76%)
26–50%	5 (10%)
51–75%	0 (0%)
76–100%	0 (0%)
Unknown	1 (2%)
*N (%) of patients with axSpA referred to nurse practitioner for follow-up*	
0%	17 (34%)
1–25%	9 (18%)
26–50%	11 (22%)
51–75%	4 (8%)
76–100%	8 (16%)
Unknown	1% (2%)
*N (%) of patients with axSpA in which the following clinical assessment were performed during follow-up*	
X-SI	75% [18–100%]
X-spine	65% [15–100%]
MRI-SI	20% [10–40%]
MRI-spine	5% [0–11%]
Bone density scan	20% [10–40%]
Serum Vitamin D	28% [5–56%]

Values are presented in *n* (%) or median [IQR]. *axSpA* axial spondyloarthritis, *e.g.* for example, *X-SI* radiograph of the sacroiliac joints, *X-spine* radiograph of the spine, *MRI-SI* magnetic resonance imaging of the sacroiliac joints, *MRI-spine* magnetic resonance imaging of the spine

In axSpA patients with stable low disease activity, disease activity was assessed a median of once a year, and in case of high disease activity, it was assessed four times a year. CRP was most often used by rheumatologists to assess disease activity, followed by BASDAI and ASDAS (82%, 38% and 28% resp.). 28% of rheumatologists always assessed ASDAS during monitoring; in contrast, 82% of rheumatologists always measured CRP. Furthermore, 26% of rheumatologists never used ASDAS to assess disease activity. See Table 4 for frequencies of disease activity assessments during follow-up.

**Table 4:** Frequency of disease activity assessments during follow-up in patients with axSpA

	Always	Often	Sometimes	Never	
ASDAS	14 (28%)	8 (16%)	15 (30%)	13 (26%)	
BASDAI	19 (38%)	12 (24%)	13 (26%)	6 (12%)	
CRP	41 (82%)	5 (10%)	3 (6%)	1 (2%)	
*Disease activity cut-off levels or improvement scores used to decide on discontinuation of bDMARDs*					
ASDAS ≤ 1.1 improvement					6 (12%)
ASDAS ≥ 2.1					3 (6%)
BASDAI ≤ 2 improvement					6 (12%)
BASDAI ≥ 4					5 (10%)
Not based on established (change in) disease activity assessment					30 (60%)

Values are presented in *n* (%). *axSpA* axial spondyloarthritis, *ASDAS* axial spondyloarthritis disease activity score, *BASDAI* bath ankylosing spondylitis disease activity index, *CRP* C-reactive protein, *bDMARDs* biological disease-modifying antirheumatic drugs

#### Treatment

**Fig. 2:**
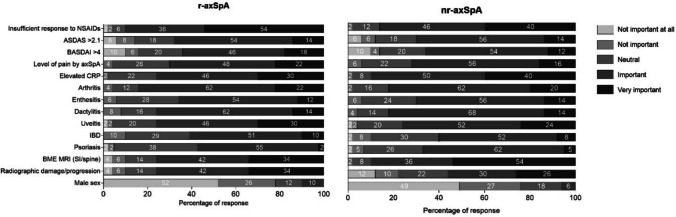
Level of importance of patient and disease characteristics when starting bDMARDs in patients with r-axSpA and nr-axSpA according to rheumatologists

A median of 60% of patients with axSpA were treated with a full dose of NSAIDs, which decreased to 20% after initiating bDMARD therapy. When deciding to initiate a bDMARD, rheumatologists showed a similar pattern for both r-axSpA and nr-axSpA in how they rated the importance of disease activity assessments and other axSpA-related features (Fig. 2). Notably, the level of pain was rated equally important as the ASDAS and BASDAI in deciding to initiate bDMARD therapy (Fig. 2). The majority of rheumatologists (60%) did not use proposed cut-off scores or changes in disease activity scores to assess the effectiveness of bDMARD treatment. Only 12% reported using the ASDAS clinically important improvement threshold of > 1.1 (Table 4). Regarding clinical guidelines, 68% of rheumatologists indicated that they use the EULAR/ASAS management recommendations in their daily practice, while 14% reported being not (sufficiently) familiar with these recommendations to do so.

## Discussion

This is the first survey among rheumatologists evaluating axSpA patients’ journey regarding the diagnosis, follow-up and treatment of axSpA in daily clinical practice. Despite significant progress in axSpA clinical research over the past decades and the publication of international management recommendations, our findings indicate that the translation of research insights into routine care seems suboptimal during this study period.

A notable finding from our survey is the persistent challenge rheumatologists face in diagnosing nr-axSpA. While most rheumatologists reported feeling confident diagnosing r-axSpA, approximately one in four expressed uncertainty when assessing patients suspected of having nr-axSpA. This is not surprising, given that the diagnosis of nr-axSpA has only existed for less than two decades [3]. This uncertainty may also be reflected by the fact that over half of the rheumatologists reported using the ASAS classification criteria as a help to confirm the diagnosis of nr-axSpA, despite the fact that these criteria were developed for research purposes and are not intended for diagnostic decision-making [8]. Also among patients with r-axSpA, one-third of rheumatologists reported relying on these criteria. Especially when expertise on imaging is lacking or the clinical arm of the ASAS classification criteria (where imaging is not mandatory for classification) is used, there is a potential risk for false-positive diagnosis which may result in overtreatment accompanied by potential risks [18, 19]. Moreover, in recognizing a possible axSpA patient, MRI of the SI joints has become more important since it provides objective signs of active sacroiliitis [20, 21]. SI-MRI was frequently used for nr-axSpA diagnosis (93%) but rarely for r-axSpA (20%); over half of rheumatologists reported insufficient expertise to confidently assess active sacroiliitis. Consequently, they often depend on the expertise of the radiologist, although about one in seven rheumatologists expressed doubt about the reliability of these interpretations. This concern is supported by recent findings suggesting that bone marrow edema on MRI, the key feature of active sacroiliitis, lacks specificity and can also appear in healthy individuals, postpartum women and athletes, although in these cases bone marrow edema is usually more superficial and at the anterior part of the SI joint [21–23]. In contrast, deep extensive lesions are highly specific for axSpA, and diagnostic accuracy improves when BME is interpreted together with structural changes such as erosions or fat metaplasia, while sclerosis is least specific [21, 22].

Our results reflect the struggle rheumatologists have in assessing disease activity or axial inflammation in patients with axSpA, which shows considerable variability in monitoring. CRP is routinely used and ASDAS was applied consistently by 28% and never by 26% of rheumatologists, despite its superior psychometric properties and association with radiographic progression compared to BASDAI [10, 24]. It should be noted that the validation of ASDAS in this context is largely based on data from RCTs [25–28], which does not fully reflect its performance or applicability in daily clinical practice. Rather than unequivocally indicating suboptimal care, limited or selective use of ASDAS may also reflect contextual clinical judgment, particularly in patients with complex symptom profiles including clinical interpretation of the origin of pain. Rheumatologists reported that the level of pain is often used for treatment decisions as much as validated disease activity scores (ASDAS and BASDAI). Using the level of pain or mainly patient-reported outcomes related to disease activity may not correspond accurately to underlying inflammation. This is also illustrated by the high ASDAS disease activity scores that persist in nearly half of the patients despite adequate anti-inflammatory treatment with overall low CRP [29]. Relying solely on pain during monitoring is questionable, since pain may (also) originate from non-inflammatory mechanisms, including central sensitisation [30, 31]. Recent studies show that central sensitisation can increase ASDAS in the absence of objective inflammation, underscoring the need to interpret disease activity within a biopsychosocial framework considering other causes of pain than inflammation [30–32]. Consequently, using ASDAS, combined with clinical judgment and complementary assessments, may represent an appropriate adaptation to real-world clinical complexity rather than simple non-adherence to guidelines.

In contrast with the ASAS/EULAR treatment recommendations, CRP and MRI were not consistently used to support treatment decisions initiating bDMARDs [17]. Elevated CRP was considered important by most rheumatologists, though less so in r-axSpA (76%) than in nr-axSpA (90%). Similarly, MRI findings were valued more in nr-axSpA (90%) than in r-axSpA (76%), while up to one-fourth considered MRI neutral or unimportant. Notably, although 68% of rheumatologists reported using the ASAS/EULAR management recommendations to guide daily care, our findings suggest that individual components of the recommendations are often not implemented in practice. When evaluating treatment response, most rheumatologists (60%) did not use any established (change in) disease activity assessment, and only a minority (24%) used the ASDAS clinically important improvement threshold of ≥ 1.1 or BASDAI response of ≥ 2 as is advised in the recommendations [17, 33].

Another important finding relates to the implementation of multidisciplinary care. Overlapping EMM such as IBD, psoriasis, and uveitis are common in axSpA and treatment decisions should be made also based on the presence of an EMM. Only a minority of rheumatologists reported regular multidisciplinary meetings with relevant specialists. Most relied on ad hoc consultations based on clinical judgment, despite overarching principle A of the ASAS/EULAR recommendations emphasizing the need for coordinated multidisciplinary management [14].

In most rheumatology practices in the Netherlands, nurse practitioners are involved in axSpA-patient care. Patient education by nurses typically focuses on disease information, lifestyle, and physical activity. However, education on disease prognosis occurs less frequently, possibly due to the uncertain nature of the disease for individual patients and the perception that discussing prognosis is primarily a medical responsibility, more appropriate for rheumatologists than for nurse practitioners, even though previous qualitative research highlights this as a key topic from the patient’s perspective [34]. Overall, referral to physiotherapists and education about exercise and smoking cessation appear well integrated, in line with recommendation 4 of the ASAS/EULAR guidelines [17].

Taken together, these findings highlight several implications for clinical practice. First, it is crucial to distinguish diagnosis from classification: in daily care, diagnosis should be guided by the overall clinical picture including pattern recognition, supported by objective evidence of active or previous inflammation of the sacroiliac joints and, when relevant, the spine, rather than relying solely on classification criteria. Secondly, adequate joint training of rheumatologists and radiologists, including trainees, in MRI interpretation is essential. Rheumatologists remain responsible for integrating clinical and imaging findings into the diagnostic process, but accurate and detailed radiological reporting, based on appropriate protocols and standardized terminology, is equally important. Strengthening education and collaboration between both specialties will improve diagnostic accuracy, reduce overdiagnosis, and optimize patient care. Third, monitoring of disease activity should not rely on pain alone. Although ASDAS has limitations and is strongly influenced by patient-reported outcomes in daily clinical practice, it currently remains the most validated instrument and is superior to BASDAI in predicting structural damage. In daily practice, greater emphasis should be placed on evaluating change in disease activity over time and on weighing these scores alongside objective signs of inflammation before and after starting b/tsDMARDs. This will improve expectation management, ensuring more appropriate treatment decisions, and ultimately optimize patient outcomes.

Future research should use qualitative and mixed-methods designs to better understand diagnostic reasoning, MRI interpretation in relation to axSpA inflammation, and disease activity monitoring in routine axSpA care. International real-life studies across different healthcare systems are needed to improve generalizability and to explore how organizational factors, referral pathways, and access to imaging modalities, especially MRI, influence clinical practice. Finally, comparing evidence from clinical trials and guideline recommendations with routine clinical care may help clarify potential gaps between scientific evidence and real-world implementation.

Strengths of this study include its being the first survey to assess the daily clinical practice of rheumatologists regarding axSpA, the use of exclusively multiple-choice questions allowing for quantitative analysis, and a representative sample of rheumatologists from various hospital types and regions.

This study has several limitations. There was a time gap between data collection and this manuscript due to the COVID period and limited personnel availability. However, the identified challenges, such as diagnostic uncertainty, MRI interpretation, and disease activity monitoring, remain highly relevant in current daily axSpA care. The survey preceded the latest ASAS/EULAR recommendation update, but we do not expect this to have substantially influenced the results, as the updates in the recommendations were limited. In the past few years, ASAS also specifically promoted not to use the classification criteria for diagnosis. Therefore, currently the number of rheumatologists using the classification criteria may be lower. Furthermore, the survey was not validated and relied on multiple-choice questions, which may have limited the nuance of the responses but enabled broad quantitative analysis. Also, not all components of the 2022 ASAS/EULAR recommendations were included, potentially missing valuable additional information [14, 17]. Furthermore, the survey was administered through face-to-face interviews conducted by employees of Novartis Netherlands. Interviewers did not receive standardized training, potentially introducing reporting bias. At the same time, interviewers primarily read questions verbatim and recorded responses without systematic probing. This study had no commercial objectives. Novartis had no role in data analysis, interpretation of the results, or manuscript preparation. Another limitation is that no clear answers were obtained from rheumatologists on why the ASDAS is not used in daily practice. We can only hypothesize rheumatologists in daily clinical practice experience a lack of sensitivity to change to a low disease activity state or remission, since data showed that ASDAS remains high in almost 50% of patients treated with bDMARDS [29]. Furthermore, the 2019 Update of the American College of Rheumatology/Spondylitis Association of America/Spondyloarthritis Research and Treatment Network Recommendations for the treatment of AS and nr-axSpA was not considered to ensure consistency with European standards [35].

## Conclusion

The results of this survey highlight a gap between clinical research findings from experts in the field including results from RCTs which are translated to international recommendations, and their implementation in routine care at the time of data collection. This gap is particularly evident in diagnosis (especially of nr-axSpA), monitoring disease activity, treatment evaluation and interdisciplinary collaboration. The limited use of ASDAS suggests that rheumatologists are unsure about its value in daily clinical practice, but this should not be interpreted as a simple gap in care. Selective or contextual use of ASDAS may reflect appropriate clinical judgment, particularly in patients with complex symptom profiles including clinical interpretation of the origin of pain. Until now ASDAS is still the best disease-activity assessment we have. Rather than promoting wider ASDAS adoption per se, improving understanding of what the ASDAS captures besides inflammation and promoting use of clinically relevant change instead of cut-off values for disease activity may help for better implementation. Furthermore, improving imaging expertise among rheumatologists and in rheumatology training, as well as promoting interdisciplinary collaboration, will be crucial to enhance implementation.

## Supplementary Information

Below is the link to the electronic supplementary material.

ESM 1 (DOCX 31.4 KB)
