# Supplementary material for: Challenges in diagnosing and monitoring radiographic and non-radiographic axial spondyloarthritis in daily clinical practice
Source: Clin Rheumatol. 2026 May 14;45(7):4265–76. doi: 10.1007/s10067-026-08157-0 (PMC13342332; doi:10.1007/s10067-026-08157-0)
Supplement: Supplementary file 1 — (DOCX 31.4 KB) [file 10067_2026_8157_MOESM1_ESM.docx]

**I-Journey: Insights into the care for patients with axial SpA in the Netherlands**

Using this questionnaire, we aim to gain insight into the care provided to patients with axial spondyloarthritis (axSpA) in the Netherlands. The ultimate goal is to use these insights to optimize patient care.

The questions cover the different phases of care, from referral through treatment and follow-up.

Important: some questions refer to axSpA in general, while others specifically address radiographic (r-axSpA) or non-radiographic (nr-axSpA). This is indicated in the questions.

**MODULE 1**

**First, a few general questions:**
*Please provide requested numbers/percentages based on your best possible estimate.*

1a. How long have you been working as a rheumatologist? ..... years (please specify as accurately as possible, preferably to the nearest half year).

1b. In what type of clinic do you work? (Please select what applies. If you work in multiple clinics, select the clinic where you have your largest FTE appointment)

☐ Academic hospital
☐ General (non-academic) hospital
☐ Independent treatment center

2. What is the total number of FTE rheumatologists in this clinic?

........ FTE

3. What is the total number of FTE nurse practitioners/specialist nurses in this clinic? ........ FTE

4. How many patients with **axSpA** are treated in your clinic?

☐ 0–200
☐ 201–400
☐ 401–600
☐ >600

4a. What percentage of these patients has **nr-axSpA**? (Important: the remaining percentage represents patients with r-axSpA)

☐ 0–20%
☐ 21–40%
☐ 41–60%
☐ 61–80%
☐ 81–100%

5. How often is the diagnosis of **axSpA** made in your clinic in an average month?

☐ 0–2
☐ 3–5
☐ 6–10
☐ >10

5a. What percentage of these newly diagnosed patients has **nr-axSpA**? (Important: the remaining percentage represents patients with r-axSpA)

☐ 0–20%
☐ 21–40%
☐ 41–60%
☐ 61–80%
☐ 81–100%

6. What percentage of **r-axSpA** patients treated in your clinic receives a bDMARD?

☐ 0–10%
☐ 11–20%
☐ 21–30%
☐ 31–40%
☐ 41–50%
☐ >50%

7. What percentage of **nr-axSpA** patients treated in your clinic receives a bDMARD?

☐ 0–10%
☐ 11–20%
☐ 21–30%
☐ 31–40%
☐ 41–50%
☐ >50%

**The different phases in care (referral → diagnosis → treatment → follow-up)**

**MODULE 2**

**Referral**

*The following questions concern* ***axSpA*** *in general.*

8. Please estimate the percentage of patients referred to your clinic with (suspected) **axSpA** by the following healthcare providers. (**Note**: the total should add up to 100%.)

- General practitioner: .......%
- Ophthalmologist: .......%
- Gastroenterologist: .......%
- Dermatologist: .......%
- Other: .......%

9. Please estimate the percentage of patients with the following symptom duration at the time of referral to your clinic with (suspected) **axSpA**. (**Note**: the total should add up to 100%.)

- < 3 months: .......%
- 3–6 months: .......%
- 7–12 months: .......%
- 1–2 years: .......%
- 2 years: .......%

10. What is the average time between referral and the first outpatient visit for patients with (suspected) **axSpA** referred to your clinic?

☐ < 1 week
☐ 1–2 weeks
☐ 2–6 weeks
☐ > 6 weeks

**MODULE 3**

**Diagnosis**

*The following question concerns* ***r-axSpA****.*

11. Please indicate how important you consider the presence of the following aspects in the medical history and physical examination when establishing the diagnosis of **r-axSpA:**

|  | Very unimportant | Unimportant | Neutral | Important | Very important |
| --- | --- | --- | --- | --- | --- |
| Male sex |  |  |  |  |  |
| Back pain started ≤45 years |  |  |  |  |  |
| Inflammatory back pain |  |  |  |  |  |
| Back pain improved ≥50% with NSAID |  |  |  |  |  |
| Arthritis/enthesitis/  dactylitis |  |  |  |  |  |
| Uveitis |  |  |  |  |  |
| IBD |  |  |  |  |  |
| Psoriasis |  |  |  |  |  |

*The following question concerns* ***nr-axSpA****.*

12. Please indicate how important you consider the presence of the following aspects in the medical history and physical examination when establishing the diagnosis of **nr-axSpA**:

|  | Very unimportant | Unimportant | Neutral | Important | Very important |
| --- | --- | --- | --- | --- | --- |
| Male sex |  |  |  |  |  |
| Back pain started ≤45 years |  |  |  |  |  |
| Inflammatory back pain |  |  |  |  |  |
| Back pain improved ≥50% with NSAID |  |  |  |  |  |
| Arthritis/enthesitis/  dactylitis |  |  |  |  |  |
| Uveitis |  |  |  |  |  |
| IBD |  |  |  |  |  |
| Psoriasis |  |  |  |  |  |

*The following question concerns* ***r-axSpA****.*

13. In patients in whom you established the diagnosis r-axSpA, for what estimated percentage did you use the following additional investigations to establish the diagnosis? (The total may exceed 100%.)

- Pelvic X-ray: ......%
- MRI of the sacroiliac joints: ......%
- HLA-B27 testing: ......%
- CRP/ESR: ......%

*The following question concerns* ***nr-axSpA****.*

14. In patients in whom you established the diagnosis **nr-axSpA**, for what estimated percentage did you use the following additional investigations to establish the diagnosis? (The total may exceed 100%.)

- Pelvic X-ray: ......%
- MRI of the sacroiliac joints: ......%
- HLA-B27 testing: ......%
- CRP/ESR: ......%

*The following questions concern* ***axSpA*** *in general.*

15. Do you have access to a musculoskeletal radiologist for the evaluation of axSpA imaging?

☐ Yes
☐ No

16. For what percentage of MRI scans performed to evaluate for sacroiliitis are you **uncertain** about the accuracy of the radiology report? ......... %

17. Do you feel that you have sufficient expertise to accurately assess the presence of active and/or previous sacroiliitis on MRI?

☐ Yes
☐ No

18. When establishing the diagnosis of **r-axSpA**: (Please select one answer.)

☐ I use the modified New York criteria for r-axSpA
☐ I use the ASAS classification criteria for axSpA
☐ I do not use formal criteria

19. When establishing the diagnosis of **nr-axSpA**: (Please select one answer.)

☐ I use the ASAS classification criteria for axSpA
☐ I do not use formal criteria

20. In what estimated percentage of patients do you consider but ultimately not establish the diagnosis of **r-axSpA** due to uncertainty? ...... %

21. In what estimated percentage of patients do you consider but ultimately not establish the diagnosis of **nr-axSpA** due to uncertainty? ...... %

**MODULE 4**

**Treatment**

*The following questions concern* ***axSpA*** *in general.*

22. What percentage of your **axSpA** patients do you refer to a rheumatology nurse or nurse practitioner for education after establishing the diagnosis?

☐ 0%
☐ 1–25%
☐ 26–50%
☐ 51–75%
☐ 76–100%

22a. If you answered 1–100% to question 22:

Which topics are addressed during this education? (Please select all that apply.)

☐ Explanation of the disease
☐ Prognosis
☐ Exercise therapy and sports
☐ Lifestyle e.g., smoking and nutrition

23. What percentage of your **axSpA** patients do you refer to a physiotherapist for active exercise therapy after establishing the diagnosis?

☐ 0%
☐ 1–25%
☐ 26–50%
☐ 51–75%
☐ 76–100%

24. What percentage of patients you have diagnosed with **axSpA** use the maximum daily dose of an NSAID? ...... %

**MODULE 5**

*The following question concerns* ***r-axSpA****.*

25. Please indicate to what extent the following patient and disease characteristics contribute to your decision to initiate a bDMARD in patients with **r-axSpA**:

|  | Very unimportant | Unimportant | Neutral | Important | Very important |
| --- | --- | --- | --- | --- | --- |
| Insufficient response to NSAIDs |  |  |  |  |  |
| ASDAS >2.1 |  |  |  |  |  |
| BASDAI>4 |  |  |  |  |  |
| Level of pain by axSpA |  |  |  |  |  |
| Elevated CRP |  |  |  |  |  |
| Arthritis |  |  |  |  |  |
| Enthesitis |  |  |  |  |  |
| Dactylitis |  |  |  |  |  |
| Uveitis |  |  |  |  |  |
| IBD |  |  |  |  |  |
| Psoriasis |  |  |  |  |  |
| BME MRI (SI/spine) |  |  |  |  |  |
| Radiographic damage/progression |  |  |  |  |  |
| Male sex |  |  |  |  |  |

*The following question concerns* ***nr-axSpA****.*

26. Please indicate to what extent the following patient and disease characteristics contribute to your decision to initiate a bDMARD in patients with **nr-axSpA:**

|  | Very unimportant | Unimportant | Neutral | Important | Very important |
| --- | --- | --- | --- | --- | --- |
| Insufficient response to NSAIDs |  |  |  |  |  |
| ASDAS >2.1 |  |  |  |  |  |
| BASDAI>4 |  |  |  |  |  |
| Level of pain by axSpA |  |  |  |  |  |
| Elevated CRP |  |  |  |  |  |
| Arthritis |  |  |  |  |  |
| Enthesitis |  |  |  |  |  |
| Dactylitis |  |  |  |  |  |
| Uveitis |  |  |  |  |  |
| IBD |  |  |  |  |  |
| Psoriasis |  |  |  |  |  |
| BME MRI (SI/spine) |  |  |  |  |  |
| Radiographic damage/progression |  |  |  |  |  |
| Male sex |  |  |  |  |  |

**MODULE 6**

*The following questions concern* ***axSpA*** *in general.*

27. What percentage of axSpA patients on bDMARD continue to use a full dose of NSAIDs? ...... %

28. Based on which of the following criteria do you decide that bDMARD is ineffective in patients with **axSpA**? (Please select one answer.)

☐ If the ASDAS has not improved sufficiently (<1.1 points)
☐ If the ASDAS after treatment remains ≥1.3 or 2.1
☐ If the BASDAI has not improved sufficiently (<2 points)
☐ If the BASDAI after treatment remains ≥4
☐ I do not determine bDMARD ineffectiveness based on a predefined (change in) disease activity measure

**Follow-up**

*The following questions concern* ***axSpA*** *in general.*

29. On average, how often do you assess disease activity in patients with active **axSpA**?

...... times per year

30. On average, how often do you assess disease activity in patients with inactive **axSpA**?

...... times per year

31. Please indicate how often you use each of the following disease activity measures in patients with active **axSpA**:

a. ASDAS: always / often / sometimes / never
b. BASDAI: always / often / sometimes / never
c. CRP: always / often / sometimes / never

32. In your clinic, are there regular multidisciplinary consultation and/or joint outpatient clinics with the following specialties? (Please select all that apply; combinations of dermatologist, gastroenterologist and/or ophthalmologist are possible)

☐ Dermatologist
☐ Gastroenterologist
☐ Ophthalmologist
☐ No regular multidisciplinary consultation and/or joint outpatient clinic

33. What percentage of your **axSpA** patients are seen in the nurse practitioner clinic as part of care substitution?

☐ 0%
☐ 1–25%
☐ 26–50%
☐ 51–75%
☐ 76–100%

34. What percentage of your **axSpA** patients do you refer at any point during follow-up to a multidisciplinary rehabilitation program?

☐ 0%
☐ 1–25%
☐ 26–50%
☐ 51–75%
☐ 76–100%

35. In what percentage of your **axSpA** patients do you perform the following additional investigations during follow-up? (The total may exceed 100%.)

- X-ray sacroiliac joints: ........%
- X-ray spine: ........%
- MRI sacroiliac joints: ........%
- MRI spine: ........%
- Bone mineral density measurement: ........%
- Vitamin D measurement: ........%

36. Do you follow the 2016 ASAS–EULAR treatment recommendations for axSpA in the treatment and follow-up of axSpA patients?

☐ Yes
☐ No
☐ Unknown; you are not sufficiently familiar with these recommendations
